# Significance of multi-task deep learning neural networks for diagnosing clinically significant prostate cancer in plain abdominal CT

**DOI:** 10.3389/fonc.2025.1543230

**Published:** 2025-05-02

**Authors:** Yujun Geng, Xinlei Zhang, Ming Zhang, Jingwen Li, Meng Yang, Junzhang Tian, Xiaofen Ma

**Affiliations:** ^1^ Department of Nuclear Medicine, The Affiliated Guangdong Second Provincial General Hospital of Jinan University, Guangzhou, China; ^2^ Department of Nuclear Medicine, Meizhou People’s Hosptal (Meizhou Academy of Medical Sciences), Meizhou, China

**Keywords:** prostate cancer, multi-task deep learning, machine learning, neural networks, computed tomography

## Abstract

**Objective:**

Early detection and timely surgical intervention are crucial in reducing mortality rates associated with clinically significant prostate cancer (csPCa). Currently, clinical diagnostics primarily depend on magnetic resonance imaging (MRI) and nuclear medicine, with the potential diagnostic value of abdominal computed tomography (CT) remaining underexplored. This study aims to evaluate the effectiveness of multi-task deep learning neural networks in identifying early-stage prostate cancer using CT scans.

**Methods:**

In this study, we enrolled 539 patients from the Department of Radiology (N=461) and Nuclear Medicine (N=78). We utilized a multi-task deep learning network model (MTDL), based on the 3DUnet architecture, to segment and analyze the collected abdominal plain CT images. The predictive performance of this model was compared with a radiomics model and a single-task deep learning model using ResNet18. A diagnostic nomogram was then developed using the multi-task deep learning approach, incorporating prediction results and PSAD, age. The diagnostic performance of the different models was evaluated using the receiver operating characteristic (ROC) curve and the area under the curve (AUC).

**Results:**

The 461 patients from the Department of Radiology were divided into training and test sets at a ratio of 6:4, while the patients from the Department of Nuclear Medicine formed the validation set. Our MTDL nomogram demonstrated AUCs of 0.941 (95% confidence interval [CI]: 0.905valceedi 0.912 (95% CI: 0.904valceedi and 0.932 (95% CI: 0.883valceed in the training, test, and validation cohorts, respectively. This study indicates that combining abdominal CT with a multi-task neural network model effectively diagnoses csPCa, offering superior diagnostic performance compared to clinical models. Additionally, the multi-task neural network model outperformed both the single-task neural network model and the radiomics model in diagnostic accuracy.

**Conclusion:**

Our study demonstrated that the MTDL nomogram can accurately predict the presence of prostate cancer using abdominal CT scans, offering significant value for the early diagnosis of prostate cancer.

## Introduction

Prostate cancer (PCa) is one of the most prevalent malignant tumors worldwide, ranking highest in incidence among male cancers. It accounts for approximately 11% of all cancer-related deaths, second only to lung and bronchial cancer (1). Early-stage prostate cancer often progresses silently, with noticeable symptoms typically appearing only at advanced stages. Therefore, early detection and timely surgical intervention are essential for effective management of the disease (2).

Currently, prostate-specific membrane antigen density (PSAD) is widely used for screening and diagnosing prostate cancer (3, 4). However, this serological marker has significant limitations. Firstly, elevated PSAD levels are also observed in patients with chronic prostatitis and benign prostatic hyperplasia (BPH) (5, 6). Secondly, many early-stage prostate cancer patients do not exhibit increased PSA values or significant gland enlargement (7). Magnetic Resonance Imaging (MRI) is commonly employed in diagnosing clinically significant prostate cancer (csPCa) (8, 9). However, it has drawbacks such as high costs and observer variability. Additionally, studies indicate that MRI has low diagnostic accuracy for lesions in the prostate’s central and transitional zones (10, 11). With advancements in molecular imaging, PET/CT has become crucial for disease diagnosis and treatment selection, offering high accuracy (12–14). Nevertheless, the preparation cost of nuclear medicine drugs used in PET/CT is relatively high. Furthermore, the technique requires high liver and kidney function from patients, limiting its widespread application.

CT is one of the most widely used medical imaging techniques today, offering significantly lower examination costs compared to MRI and PET/CT. However, its limited density and soft tissue resolution make it challenging to detect cancerous lesions within the prostate gland. Additionally, benign prostate conditions like chronic prostatitis and benign prostatic hyperplasia can also cause gland enlargement (7, 15), making CT less common in clinical practice for prostate cancer diagnosis. With advancements in artificial intelligence and radiomics (16), new image recognition techniques have emerged, such as diagnosing benign and malignant lung nodules (17) and analyzing tumor microenvironments (18), These developments make detecting prostate cancer via abdominal CT scans a possibility. If histological differences exist between prostate cancer and other benign glandular tissues, they might be difficult for the naked eye to discern but could potentially be detected by computer vision. This study aims to explore this potential.

Deep learning neural networks are at the forefront of disease prediction, demonstrating robust capabilities in image classification (17, 18), segmentation (19), synthesis (20, 21), and detection across numerous studies (22). These predictive models have shown potential to outperform traditional radiomics-based models (23–25). However, current single-task models typically use convolutional neural networks (CNNs) to extract image features, followed by predictions through multi-layer linear models, often without requiring segmentation masks for regions of interest. Without these masks, models may focus on irrelevant areas, leading to errors and poor generalization (24). Additionally, the black-box nature of deep learning limits reproducibility, resulting in unstable outcomes. Subtle changes can severely degrade performance, causing single-task models to achieve high accuracy on internal datasets but suffer from overfitting and reduced accuracy on external datasets. Increasing sample sizes could mitigate this, but the unique and scarce nature of medical data makes it challenging. Recently, multi-task deep learning models have emerged, performing disease prediction alongside segmentation of regions of interest (22, 26). This approach allows the segmentation task to guide the CNN in extracting relevant features, while also retaining information outside the region of interest to enhance prediction accuracy. However, the effectiveness of multi-task deep learning for predicting prostate cancer using CT has not yet been validated in large patient cohorts.

In this study, we aim to assess the effectiveness of multi-task deep learning in predicting prostate cancer using abdominal CT images from radiology and nuclear medicine departments. We utilized a cutting-edge multi-task prediction model based on the 3DUnet framework (27), which predicts cancer risk for patients suspected of having prostate cancer and segments the prostate gland. Additionally, we developed a diagnostic nomogram based on multi-task deep learning to enhance the accuracy and generalizability of clinically significant prostate cancer predictions. We will also compare the performance of multi-task deep learning models, single-task deep learning models, and radiomics models in diagnosing clinically significant prostate adenocarcinoma using abdominal CT.

## Materials and methods

This retrospective study was approved by the institutional review boards of The Affiliated Guangdong Second Provincial General Hospital of Jinan University, and the requirement for obtaining informed consent was waived.

### Participants

**Figure 1:**
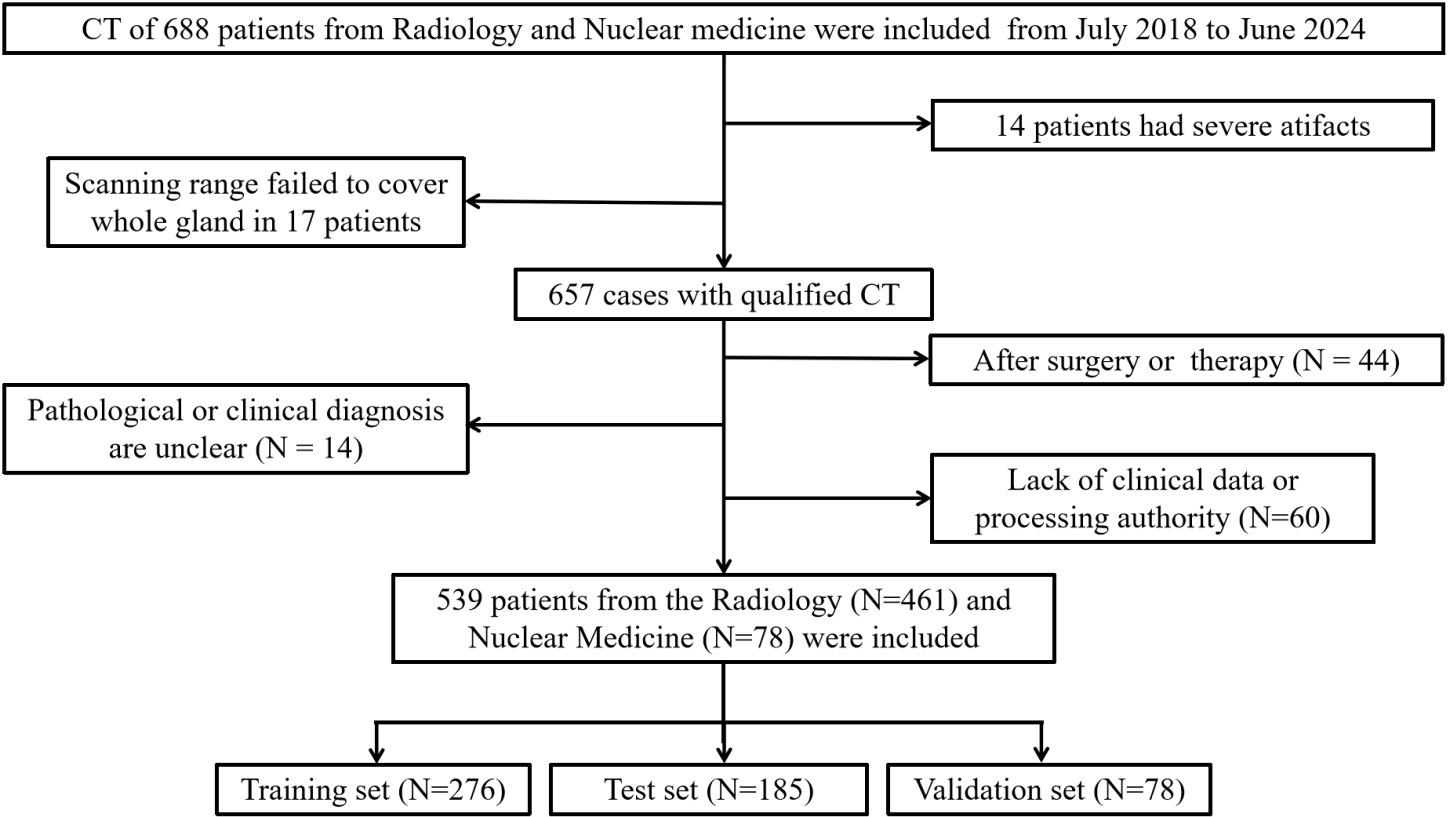
Flow diagram of the inclusion and exclusion of cases.

From July 2017 to June 2024, we included abdominal CT images from 578 patients in the Department of Radiology and PET/CT images from 110 patients in the Department of Nuclear Medicine at Guangdong Second Provincial General Hospital (**Figure 1**). Each patient had definitive pathological biopsy results confirmed in subsequent clinical follow-ups. For those diagnosed with prostate cancer, a Gleason score was required, with scores of 7 or higher indicating clinically significant prostate cancer. The inclusion criteria were: (1) Elevated PSA levels, enlarged prostate volume, or abnormal nodule signs on MRI, ultrasonography, or digital rectal examination; (2) A time interval of less than 4 weeks between CT and biopsy. The exclusion criteria were: (1) CT scans performed after surgery or other treatments; (2) Severe disease in other organs with metastasis; (3) Poor image quality or presence of significant metal or motion artifacts; (4) Incomplete clinical data or unavailable images and laboratory data (**Supplementary Table S1**).

Finally, 461 patients from Radiology and 78 patients from Nuclear Medicine were enrolled in this study. Patients from Radiology were randomly divided into a training set (n=276) and an test set (n=185) with a 6:4 ratio, while patients from Nuclear Medicine (n=78) were used as validation set and used merely for evaluation purpose.

### PET/CT image scanning and results acquisition

Patients from Radiology underwent conventional CT (uCT960+ scanner; United Imaging Healthcare, Shanghai, China) with 120 kV in our research. The scanning range included the region from theapex of the diaphragm to the mid-thigh for the CT. A spiral CT scan (tube voltage, 120 kV; tube current, 150–300 mA; layer thickness and reconstruction layer thickness, 3 mm) was performed with the 512×512 matrix; field of view (FOV) = 512×512 mm2; slice thickness = 1.0 mm; and 250 transverse sections without gaps. CT images were obtained with 0.5 mm collimation and reconstructed into axial images every 2.0 mm on a 512×512 matrix using iterative reconstruction algorithms associated with each vendor’s CT scanner. We collected these CT images at our local center’s PACS system.

Patients from Nuclear Medicine underwent conventional PET/CT (uMI780 scanner; United Imaging Healthcare, Shanghai, China) with 120 kV in our research. The scanning range included the region from the skull top to the mid-thigh for the PET/CT. A spiral CT scan (tube voltage, 120 kV; tube current, 100–500 mA; layer thickness and reconstruction layer thickness, 3.0 mm) was performed first, and the radiotracer 18F-FDG (3.7 MBq/kg body mass) was intravenously injected. After 60 minutes, PET images (4 beds, each scanned for 2.5 minutes, with a 150×150 matrix, a layer thickness of 2.68 mm) were acquired. PET image attenuation correction and iterative reconstruction were performed using CT data, with 2 iterations and a subset of 20. PET reconstruction was performed using an ordered subset expectation maximization (OSEM) and setting a spectrum of parameters, for instance, VUE Point FX module (United Imaging Healthcare), 3 iterations, 24 subsets, matrix 192×192, slice thickness of 3.27 mm, pixel size of 3.65×3.65×3.27 mm3 with a filter (6.4 mm), and all necessary correction methods including attenuation and scatter correction.

### Imaging segmentation and feature extraction

**Figure 2:**
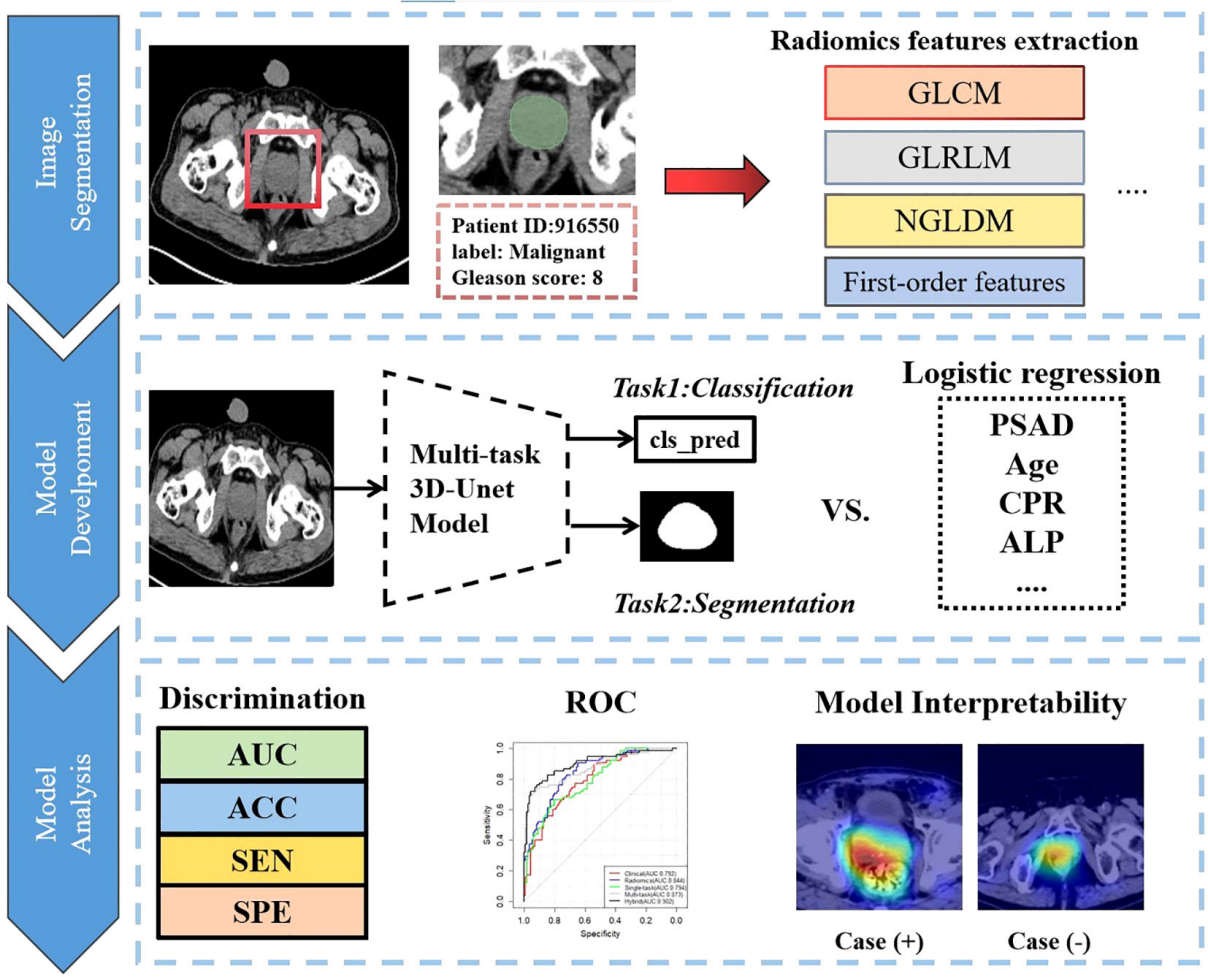
Overall analysis flow chart of this research.

We imported the CT images of all patients into the open-source software package 3D-slicer software (https://www.slicer.org) (28). Two experienced radiologists, each with over seven years in the Radiology department, used the software to delineate the entire prostate as the region of interest (ROI). Any discrepancies were resolved through discussion between the physicians. Using the open-source Python package PyRadiomics, we extracted a total of 1,155 radiomic features and assessed the correlation between the two patient groups. These features included 28 morphological features, 20 original first-order features, 24 gray-level co-occurrence matrix (GLCM) features, 14 gray-level dependence matrix (GLDM) features, 16 gray-level run-length matrix (GLRLM) features, 16 gray-level size-zone matrix (GLSZM) features, 5 neighboring gray-level dependence matrix (NGLDM) features, and 1,032 features derived from wavelet and Laplacian of Gaussian (LoG) transformations. For more details, please refer to **Figure 2**.

### Feature selection and calculate PSA density

We used the Lasso regression method to select features and build a multiple linear regression model. Feature selection and model construction were performed on an independent training set and subsequently validated using both test and validation sets. A total of 45 groups of radiomic features were selected for model construction in this study.

PSAD was calculated by dividing the total PSA (ng/ml) by the prostate volume (ml). This study used the prolate ellipsoid formula based on CT images to calculate prostate volume, measuring three diameters directly on the CT image and using the formula: width × height × length × π/6 (29). Prostate volume measurements were also conducted using the 3D Slicer software.

### Images preprocessing and DL model development

We selected ResNet-18 (**Supplementary Figure S3**) as our single-task neural network architecture. It includes one input convolutional layer, eight residual convolutional blocks (comprising 16 convolutional layers), and one fully connected layer. We optimized the network parameters using the Binary Cross Entropy (BCE) loss function and updated parameters with the Adam optimizer, set at a learning rate of 0.00001.

For the multi-task diagnostic prediction model, we modified the 3D-Unet (**Supplementary Figure S3**) as the foundational framework. After the main four down-sampling modules, we applied average pooling and connected the results to a multi-layer linear model for prediction. This approach allows the model to output both prediction results and the mask of the region of interest. We utilized the Tversky loss function to optimize segmentation results, setting α to 0.7 and β to 0.3 to enhance the segmentation sensitivity of our CNN model. The final loss function combines both classification and segmentation losses. We used the Adam optimizer with a learning rate of 0.00001 and a batch size of 5. The evaluation metrics include Area Under the Curve (AUC), accuracy (ACC), sensitivity (SEN), and specificity (SPE).


$$\begin{array}{c} Tversky\;loss\;\left(Lseg\right)=1-[(\sum pred\;*\;label+\alpha abe/(\sum pred\;\\ {}*\;label+\alpha a\;\left(1-label\right)\;*\;pred+\beta r\;label\;*\;\left(1-pred\right))] \end{array}$$

$$BCE\;loss\;(Lcls)=-\sum y\times log\left(y^{hat}\right)+\left(1-y\right)\times log\left(1-y^{hat}\right)$$

Ltotal = Lcls + Lseg

Before training the deep learning neural network model, we preprocessed the images and performed data augmentation to enhance the model’s generalization ability. The augmentation techniques included flipping, cropping, rotating, shifting, and sharpening. Specifically, 40% of the images were randomly selected for horizontal and vertical flipping. The cropping range was set between 0-10%, while the shift magnitude was set to ±15%. For rotation, angles were adjusted by ±30°. We employed the Gradient-weighted Class Activation Mapping (Grad-CAM) (30) technique to visualize the output features of the last convolutional layer of the deep learning network. This helps us understand the model’s focus and ensures that its predictions are primarily based on the prostate. To prevent overfitting, we added batch normalization layers after each convolutional kernel and included Dropout layers with a rate of 0.35 in the fully connected layers, which are more susceptible to overfitting. The training process is halted if the loss function decreases in the training set but not in the test and validation sets for more than 25 epochs. For details on the model training process, please see **Supplementary Figure S1**.

Subsequently, the deep learning scores were combined with clinically significant variables (P<0.05) identified through univariate logistic regression analysis to construct a Nomogram diagnostic model. Given the class imbalance in our study population—where malignant cases constituted only 34.32% of all cases, especially in the training and validation sets—we employed the SMOTE algorithm. This approach balanced the number of positive and negative samples and expanded the size of the validation set, thereby enhancing the credibility of the results.

### Statistical analysis

Continuous parameters are described using the median or mean (with range), while categorical variables are presented as frequency (with percentage). Differences between the training, internal validation, and external validation cohorts are analyzed using the Mann-Whitney test, chi-square test, or Fisher’s exact test. Univariate and multivariate logistic regression analyses are conducted using SPSS (version 26.0). All nomograms are developed based on multivariate analyses in R 4.3.0. The area under the curve (AUC), accuracy (ACC), sensitivity (SEN), specificity (SPE), and 95% confidence interval (95% CI) are calculated to compare each model. The DeLong test is employed to compare the ROC curves of the models across groups. A p-value of less than 0.05 is considered statistically significant.

## Results

### Patient clinical characteristics

The demographic and clinical characteristics of the patients are detailed in (**Table 1**). The median ages for the training, test, and validation cohorts are 70 years (range 50–89 years), 70 years (range 46–90 years), and 69 years (range 47–91 years), respectively. Across these three cohorts, there were no statistically significant differences in Age (P=0.558), tPSA (P=0.291), gland volume (P=0.917), PSAD (P=0.183), ALP (P=0.630), and CRP (P=0.862). Based on the results of pathological biopsy, clinical follow-up, or surgical procedures, the proportions of malignancy (Gleason score >= 7) in the training, test, and validation groups were 30.43% (84/276), 40.00% (74/185), and 29.49% (23/78), respectively. There were no significant differences in the distribution of benign and malignant differences among the groups (P=0.213, 0.288).

**Table 1:** Demographic and clinical characteristics of patients (N=539).

Characteristics	Training group (N=276)	Test group (N=185)	Validation group (N=78)	P-value*
***Age (years)***	70 (50-89)	70 (46-90)	69 (47-91)	0.558
***tPSA(ng/ml)***	7.63 (3.2-20.9)	7.56 (3.3-27.3)	13.5 (6.9-27.0)	0.291
***Volume(cm^3^)***	11.75 (83.8-166.2)	10.97 (79.2-162.4)	11.57 (77.6-163.3)	0.917
***PSAD(ng/ml/cm^3^)***	0.12 (0.06-0.28)	0.12 (0.06-0.35)	0.18 (0.10-0.68)	0.183
ALP(U/L)*	70.5 (58.0-86.0)	71.0 (57.0-86.0)	74.5 (57.0-91.0)	0.630
CRP(mg/L)*	3.55 (0.60-13.83)	3.10 (0.50-15.31)	3.15(0.52-9.71)	0.862
***Pathology***				0.213
BPH*	54 (19.57%)	57 (30.81%)	30 (38.46%)	
***Prostatitis***	8 (2.90%)	7 (3.78%)	14 (17.95%)	
BPH & Prostatitis*	128 (46.38%)	43 (23.24%)	11 (14.10%)	
**Gleason Score 6**	2 (0.72%)	4 (2.16%)	0 (0.00%)	
Gleason Score 7*	20 (7.25%)	28 (15.14%)	3 (3.85%)	
Gleason Score 8*	29 (10.51%)	22 (11.89%)	6 (7.69%)	
Gleason Score 9*	20 (7.25%)	17 (9.19%)	11 (14.10%)	
Gleason Score 10*	15 (5.43%)	7 (3.78%)	3 (3.85%)	

Normally distributed continuous variables are represented as mean ± variance; abnormally distributed continuous variables are represented as median (interquartile range, IQR); categorical variables are represented as the number of cases (percentage, %); tPSA, total prostate-specific antigen; BPH, Benign prostate hyperplasia; ALP, Alkaline phosphatase; CRP, C-reactive protein; PSAD, prostate-specific antigen density; Gleason score is greater than or equal to 7, we consider it to be a patient with clinically significant prostate cancer (csPCa), otherwise it is benign; P-value less than 0.05 was considered statistically significant. The bold text represents the row and column names in the tables. If a number within the table is bolded, it indicates that the differences between the data are of clinical statistical significance.

### Performance of clinical model, radiomics model, single-task deep learning model and multi-task deep learning

**Figure 3:**
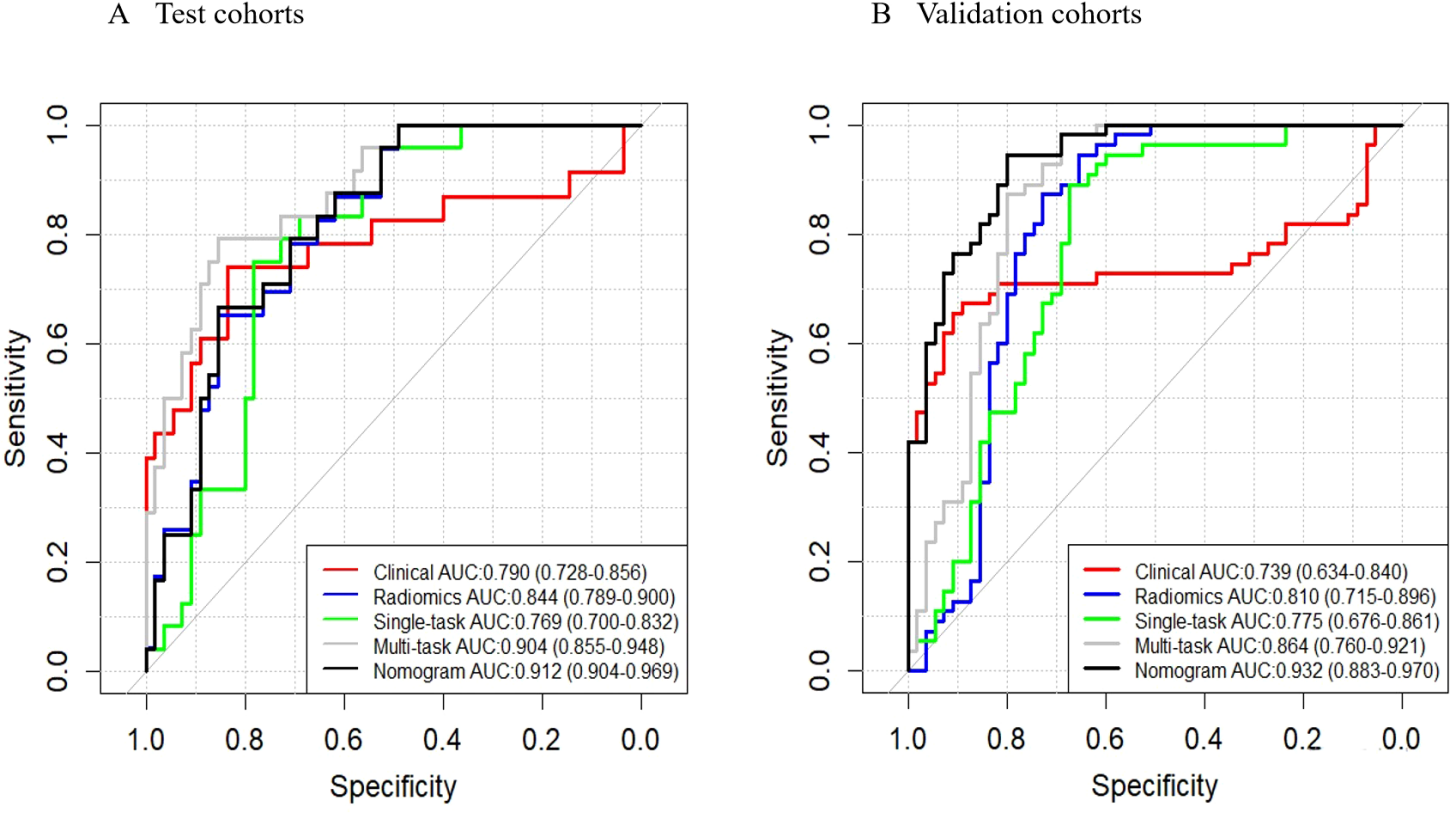
ROC curves. These curves illustrate the performance of different models, including the clinical model, radiomics model, single-task deep learning model, multi-task deep learning model, and nomograms, in both the test **(A)** and validation **(B)** sets.

We established a clinical prediction model based on 6 clinical radiological features and a radiomics prediction model based on 45 radiomics features. The AUC, accuracy, sensitivity, specificity of Clinical model (**Table 2**) were 0.790 (95% CI: 0.782-0.856), 0.708 (95% CI: 0.641-0.750), 0.770 (95% CI: 0.670-0.864), 0.667 (95% CI: 0.579-0.754) in test set and 0.739 (95% CI: 0.634-0.840), 0.782 (95% CI: 0.705-0.859), 0.655 (95% CI: 0.529-0.780), 0.909 (95% CI: 0.833-0.985) in validation set (**Figure 3**).

**Table 2:** Univariate logistic regression analysis (clinical model) for csPCa prediction on the training, test and validation cohorts.

Characteristics	Training group		Test group		Validation group	
	OR (95%CI)	P value	OR (95%CI)	P value	OR (95%CI)	P value
tPSA	1.52 (0.14-1.17)	0.327	2.62 (1.02-4.75)	0.002	1.03 (0.58-2.99)	0.262
Volume	0.07 (0.01-0.52)	0.822	1.44 (0.79-2.19)	<0.001	0.34 (0.02-1.23)	0.448
PSAD	7.11 (2.72-11.87)	0.002	0.99 (0.59-2.72)	0.224	0.91 (0.13-3.67)	0.394
Age	0.58 (0.22-0.96)	0.002	0.41 (0.04-0.81)	0.035	0.69 (0.01-1.43)	0.055
ALP	1.03 (0.20-2.07)	0.026	0.53 (0.07-1.23)	0.104	1.61 (0.12-4.24)	0.171
CRP	0.01 (0.00-0.36)	0.986	0.03 (0.00-0.53)	0.889	0.07 (0.00-0.67)	0.805

tPSA, Total prostate-specific antigen; ALP, Alkaline phosphatase; CRP, C-reactive protein; PSAD, Prostate-specific antigen density; OR, Odds ratio, 95% CI, 95% confidence interval (2.5–97.5%); P value less than 0.05 was in bold.

The AUC, accuracy, sensitivity, specificity of Radiomics model were 0.844 (95% CI: 0.789-0.900, P = 0.202), 0.746 (95% CI: 0.619-0.730), 0.905 (95% CI: 0.839-0.972), 0.649 (95% CI: 0.560-0.738) in test set and 0.810 (95% CI: 0.715-0.896, P = 0.292), 0.800 (95% CI: 0.725-0.875), 0.873 (95% CI: 0.785-0.961), 0.727 (95% CI: 0.610-0.845) in validation set (**Figure 3**).

The AUC, accuracy, sensitivity, specificity of Single-task deep learning model were 0.769 (95% CI: 0.700-0.832, P = 0.662), 0.654 (95% CI: 0.596-0.708), 0.905 (95% CI: 0.839-0.972), 0.487 (95% CI: 0.394-0.580) in test set and 0.775 (95% CI: 0.676-0.861, P = 0.573), 0.782 (95% CI: 0.705-0.859), 0.891 (95% CI: 0.809-0.973), 0.673 (95% CI: 0.549-0.797) in validation set (**Figure 3**).

The AUC, accuracy, sensitivity, specificity of Multi-task deep learning model were 0.904 (95% CI: 0.855-0.948, P = 0.006), 0.865 (95% CI: 0.834-0.928), 0.730 (95% CI: 0.629-0.831), 0.955 (95% CI: 0.916-0.994) in test set and 0.864 (95% CI: 0.760-0.921, P = 0.042), 0.836 (95% CI: 0.767-0.906), 0.982 (95% CI: 0.947-1.000), 0.691 (95% CI: 0.569-0.813) in validation set (**Figure 3**).

### Establishment of prediction nomogram model

Given that single-task deep learning network models (Resnet-18) underperform compared to multi-task deep learning network models in diagnosis, we will use the predictions from the multi-task deep learning model for further analysis in our subsequent discussions. In this study, none of the parameters we examined showed significant correlations with the final pathological results in the training, testing, and validation sets. However, in the independent training set, PSAD, Age, and ALP demonstrated independent predictive factor (P<0.05) in predicting the csPCa (**Table 2**). However, since the OR value for ALP is only 1.03, its impact on the results is minimal. To mitigate the risk of overfitting, we included PSAD, Age, and MTDL as factors in a multivariable logistic regression model to construct a hybrid model.

**Figure 4:**
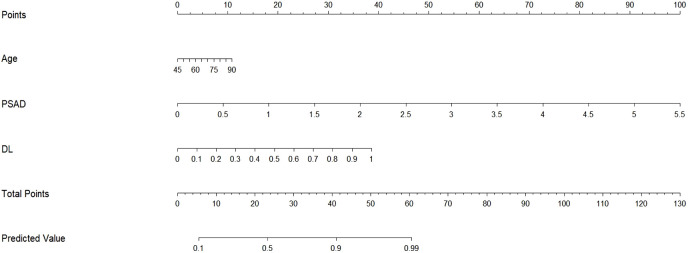
Nomogram for hybrid model prediction of clinically significant prostate cancer. We developed a nomogram using the training set, incorporating Age, PSAD, and the predicted probabilities from the multi-task deep learning model to estimate the likelihood of csPCa. To use the nomogram, first determine the predicted probability from the neural network model for the patient and draw a vertical line to the “points” axis to find the corresponding score. Repeat this process for Age and PSAD, then sum the points from these three covariates. Finally, locate this total on the “total points” axis and draw a vertical line down to assess the probability of the patient having csPCa.

**Figure 5:**
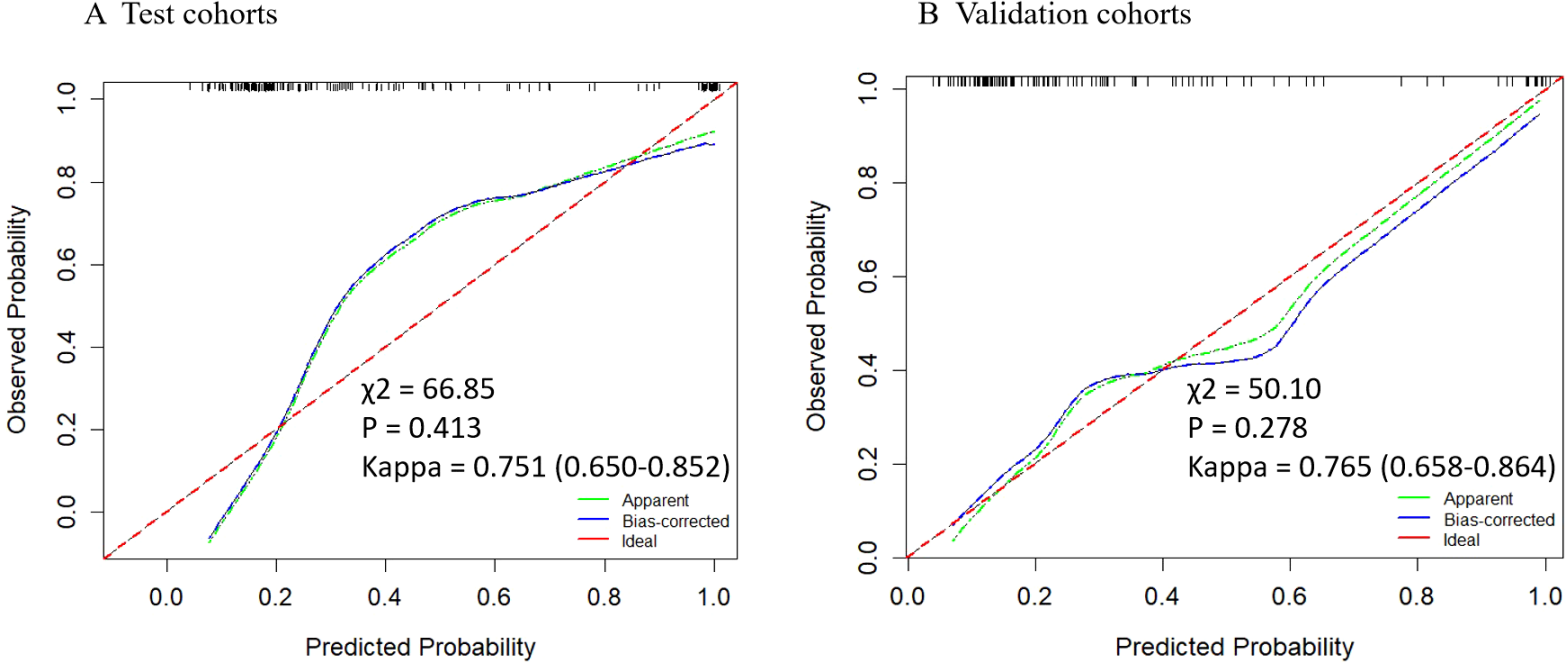
Calibration curves of the nomogram in test and validation cohorts. The y-axis represents the actual probability, while the x-axis shows the predicted probability by the nomogram. The calibration p-value is calculated using the Hosmera Lemeshow goodness-of-fit test , where a P-value greater than 0.05 indicates a good match between the actual and predicted probabilities. The kappa value at the bottom represents the results of Cohen’s consistency test (95% CI), with values greater than 0.7 indicating high consistency.

Based on the multivariate analysis, we built the prediction nomogram (**Figure 4**) with PSAD, Age, and DL. Multivariate logistic regression analysis showed that DL (B = 5.62, 95% CI: 2.74-8.55, p = <0.001), Age (B = 0.07, 95% CI: 0.01-0.14, p = 0.021), and PSAD (B = 5.13, 95% CI: 4.27-7.26, p < 0.001) were independently associated with predicting csPCa (**Table 3**). The AUC of the nomogram was 0.941 (95% confidence interval (CI): 0.905-0.970, P<0.001), 0.912 (95% CI: 0.904-0.969, P = 0.002), and 0.932 (95% CI: 0.883-0.970, P <0.001) in the training, test, and validation cohort (**Table 4**). The calibration curves (**Figure 5**) in the test and validation sets indicate that the nomogram possesses good predictive diagnostic performance. The p values of the Hosmer–Lemeshow test in the test set and the validation set were 0. 413 (χ2 = 66.85), 0.278 (χ2 = 50.10), respectively (p > 0.05). The results of the Cohen’s test indicate that the consistency between the predicted outcomes of the Nomogram model and the actual results is relatively strong with Kappa is 0.751 (0.650-0.852) and 0.765 (0.658-0.864) in the test set and the validation set.

**Table 3:** Multivariate logistic regression analysis (Hybrid model) for csPCa prediction on the training, test and validation cohorts.

Characteristics	Training group		Test group		Validation group	
	B value (95%CI)	P value	B value (95%CI)	P value	B value (95%CI)	P value
**Age**	0.07 (0.012-0.138)	**0.021**	0.03 (0.012-0.079)	0.154	0.05 (0.029-0.129)	0.229
**PSAD**	5.13 (4.266-7.255)	**<0.001**	1.12 (0.194-2.336)	**0.046**	1.50 (0.503-2.954)	**0.016**
**DL**	5.62 (2.744-8.546)	**<0.001**	7.38 (4.131-12.344)	**<0.001**	4.82 (2.049-9.996)	**0.009**

tPSA, Total prostate-specific antigen; DL, Deep learning model; PSAD, Prostate-specific antigen density; OR, odds ratio, 95% CI, 95% confidence interval (2.5–97.5%). The bold text represents the row and column names in the tables. If a number within the table is bolded, it indicates that the differences between the data are of clinical statistical significance.

**Table 4:** Predictive performance of clinical model, radiomics model, single-task deep learning model, multi-task deep learning model and hybrid model on the training, test, validation sets.

Signatures	AUC (95%CI)	ACC (95%CI)	Sensitivity (95%CI)	Specificity (95%CI)	P-value*
Training set					
Clinical	0.753 (0.682-0.824)	0.826 (0.781-0.871)	0.536 (0.423-0.638)	0.953 (0.923-0.983)	**Ref**
Radiomics	0.849 (0.802-0.898)	0.804 (0.762-0.854)	0.691 (0.600-0.798)	0.854 (0.804-0.904)	**0.011**
Single-task DLM	0.897 (0.857-0.930)	0.801 (0.750-0.845)	0.845 (0.768-0.923)	0.781 (0.723-0.840)	**<0.001**
Multi-task DLM	0.906 (0.859-0.948)	0.880 (0.855-0.928)	0.786 (0.657-0.843)	0.922 (0.884-0.960)	**<0.001**
Nomogram	0.941 (0.905-0.970)	0.891 (0.850-0.925)	0.833 (0.855-0.928)	0.917 (0.878-0.956)	**<0.001**
Test set					
Clinical	0.790 (0.728-0.856)	0.708 (0.641-0.750)	0.770 (0.670-0.864)	0.667 (0.579-0.754)	**Ref**
Radiomics	0.844 (0.789-0.900)	0.746 (0.619-0.730)	0.905 (0.839-0.972)	0.649 (0.560-0.738)	0.202
Single-task DLM	0.769 (0.700-0.832)	0.654 (0.596-0.708)	0.905 (0.839-0.972)	0.487 (0.394-0.580)	0.662
Multi-task DLM	0.904 (0.855-0.948)	0.865 (0.834-0.928)	0.730 (0.629-0.831)	0.955 (0.916-0.994)	**0.006**
Nomogram	0.912 (0.904-0.969)	0.891 (0.837-0.935)	0.824 (0.738-0.911)	0.919 (0.857-0.963)	**0.002**
Validation set					
Clinical	0.739 (0.634-0.840)	0.782 (0.705-0.859)	0.655 (0.529-0.780)	0.909 (0.833-0.985)	**Ref**
Radiomics	0.810 (0.715-0.896)	0.800 (0.725-0.875)	0.873 (0.785-0.961)	0.727 (0.610-0.845)	0.292
Single-task DLM	0.775 (0.676-0.861)	0.782 (0.705-0.859)	0.891 (0.809-0.973)	0.673 (0.549-0.797)	0.573
Multi-task DLM	0.864 (0.760-0.921)	0.836 (0.767-0.906)	0.982 (0.947-1.000)	0.691 (0.569-0.813)	**0.042**
Nomogram	0.932 (0.883-0.970)	0.873 (0.810-0.935)	0.946 (0.885-1.000)	0.800 (0.694-0.906)	**<0.001**

AUC area under the curve; CI, confidence interval; DLM, deep learning model; Delong’s test to compare the differences between AUCs while p < 0.05 was considered statistically significant. The bold text represents the row and column names in the tables. If a number within the table is bolded, it indicates that the differences between the data are of clinical statistical significance.

### Visualization of the DL signature

**Figure 6:**
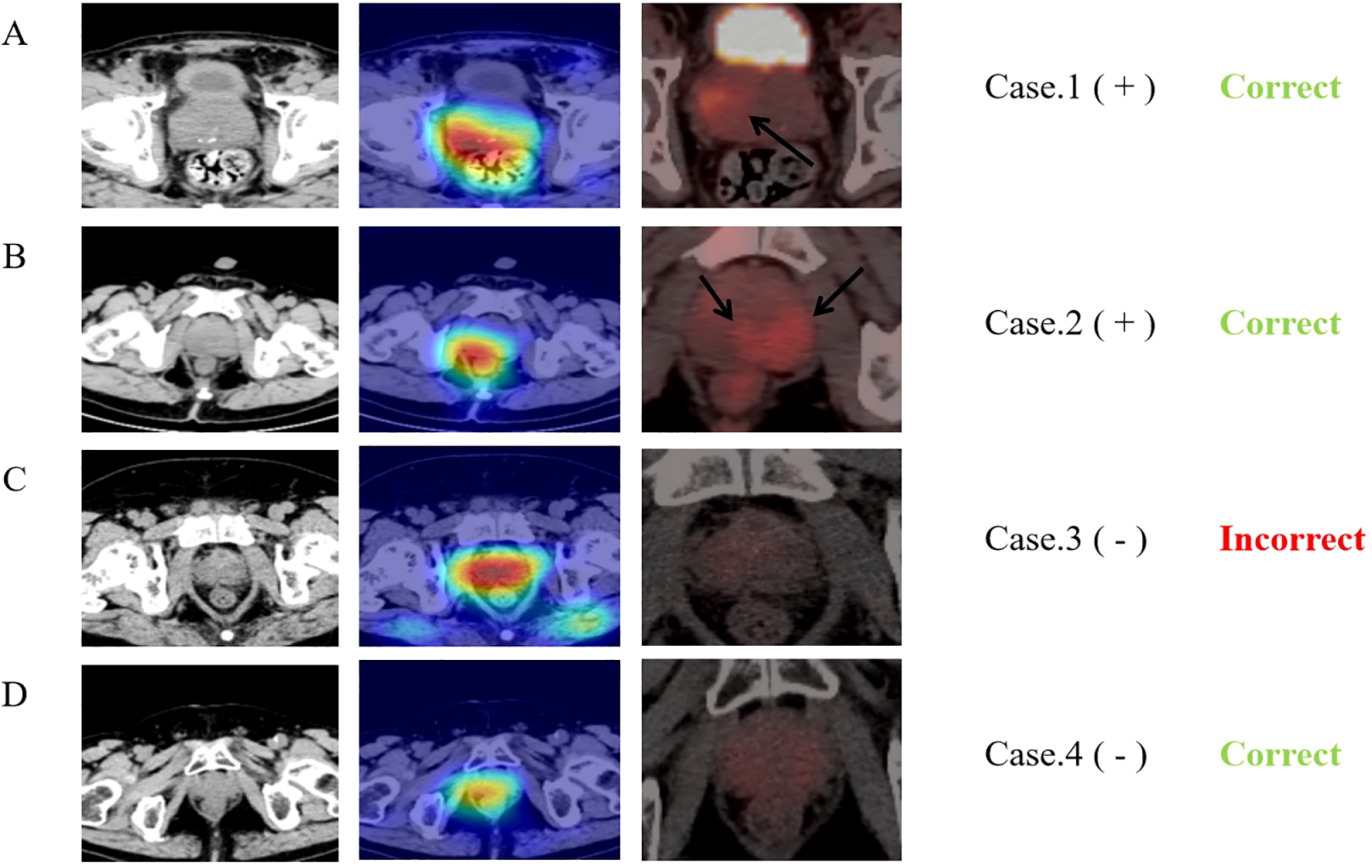
Comparison of heat maps from MTDL and PET/CT. The first column displays CT images of four typical cases and second row presents the corresponding Grad-CAM heat maps, calculated from the last downsampling convolutional layer of the multi-task model. The third row shows the PET/CT images of the same layers and “+” and “-” following the cases represent the benign or malignant diagnosis results. The last row indicates the final predictions of the neural network. The first case is an 81-year-old prostate cancer patient with a Gleason score of 9, neural network model predicts correctly and the network’s main focus area partially overlaps with the tracer concentration area in PET. The second case is a 74-year-old prostate cancer patient with a Gleason score of 9, although the neural network model predicts correctly, the network’s focus area does not overlap with the tracer concentration area in PET at all. The third case is a 60-year-old prostatitis patient, and the neural network predicts an incorrect result (malignant). The fourth case is a 68-year-old patient with chronic prostatic hyperplasia and prostatitis, and the neural network predicts correctly.

Based on the deep features of the last convolutional layer of the trained multi-task model, we performed Grad-CAM feature visualization on the test and validation sets (**Figure 6**). The darker the color, the greater the impact on the result. Notably, in the 23 malignant cases in the validation set, the neural network correctly predicted 15 cases (65.22%). Among these, 11 (47.83%) cases showed higher degree of overlap with the region of FDG tracer accumulation (Cancerous area), while 4 (17.39%) cases, although correctly predicted, had a lower degree of overlap between the two regions. Additionally, we compared the heatmaps of single-task neural networks and multi-task neural networks (**Supplementary Figure S2**). The results indicate that, compared to the single-task neural network model, the multi-task neural network model focuses more on the prostate tissue in the CT images.

## Discussion

In this study, we developed five distinct clinical diagnostic models capable of predicting benign prostate diseases and clinically significant prostate cancer (Gleason score ≥ 7) using abdominal plain CT scans. The Nomogram model, developed with training cohort data, demonstrated strong differentiation in the test and validation cohorts, achieving area under the curve values of 0.912 and 0.932, and accuracy rates of 0.891 and 0.873, respectively. These results suggest that the multi-task deep learning network model effectively assists in predicting prostate cancer lesions from abdominal plain CT scans, maintaining robust diagnostic performance even when the CT images are sourced from different departments (radiology and nuclear medicine). In prostate cancer screening and detection, this model serves as a valuable supplement to conventional ultrasound and MRI, aiding clinicians in optimizing biopsies and decision-making, and determining or eliminating the need for further biopsies. Additionally, the heatmap generated by the neural network model can approximately identify lesion locations, providing a crucial basis for clinical percutaneous biopsies and reducing unnecessary procedures. Moreover, the model we proposed is based on 3D Unet, a widely used model for medical image segmentation. Its architecture is relatively simple, with only four downsampling and four upsampling layers, offering high inference efficiency. It also has a relatively small number of parameters and moderate GPU memory requirements, making it suitable for institutions with limited computational resources.

Similarly, it’s worth noting that in our initial model training, we used Dice loss as the loss function for the neural network. This choice, along with data imbalance and other factors, led to significant overfitting. Initially, the model’s accuracy on the test and validation sets was only 0.85 and 0.83, with sensitivities of 0.74 and 0.78, which did not surpass the clinical model’s accuracies and sensitivities of 0.82, 0.76 and 0.70, 0.65 on the test and validation sets. However, after modifying the training loss function, we resolved the overfitting issue, achieving accuracy and sensitivity of 0.89 and 0.82, and 0.87 and 0.95 on the test and validation sets, respectively. This highlights the importance of selecting an appropriate training strategy to enhance model performance. This is particularly crucial for detection models of malignant lesions, where high sensitivity is essential.

Significant advancements have been made in the diagnosis and prognosis of prostate cancer, particularly through imaging techniques such as ultrasonography, MRI, and PSMA-PET/CT. Numerous studies have also focused on radiomics and deep learning. For instance, a multicenter study by Soeterik et al. (31) demonstrated a significant correlation between PSMA-PET/CT parameters—such as maximum standardized uptake value (SUVmax), total volume of PSMA (PSMA-TV), and total lesion of PSMA (TLP)—with the ISUP grading of prostate cancer and postoperative grading. In recent literature, Yi et al. (32) developed a predictive model using radiomic features of PSMA-PET/CT. By analyzing both the standard and delayed periods of PET, they successfully predicted prostate cancer lesions without PSMA tracer accumulation, achieving an AUC of 0.925. Similarly, Boschheidgen et al. (33) showed that MRI parameters, such as the length to prostatic pseudocapsule and apparent diffusion coefficient (ADC), could effectively predict the upgrading of prostate cancer lesions (from ISUP 1 to ISUP ≥ 2). The B value of the MRI grade group (mGG) was the highest. However, this study only included patients with obvious prostate cancer lesions on MRI (PI-RADS ≥ 3), indicating the need for more detailed research.

With the continuous advancement of artificial intelligence and deep learning technologies, their application in the clinical field is expanding, including clinical data processing, pathology recognition, and the generation of medical reports using natural language processing (34–36). These technologies are particularly valuable for radiologists, as they can automatically identify and summarize imaging features that are challenging or impossible for the human eye to detect (37, 38). This capability significantly reduces the workload of clinical physicians and facilitates precision medicine. However, deep learning technologies have inherent limitations, such as low interpretability and challenges in controlling training optimization, which restrict their clinical application. To address these issues, multi-task deep learning has emerged as an improvement over previous single-task methods. This approach allows neural networks to focus on specific areas within an image, increasing interpretability and enhancing generalization. Li et al. (24) demonstrated that a multi-task neural network model could effectively identify muscle invasion in bladder cancer using MRI images, achieving an AUC of 0.932. The heat map analysis showed a more concentrated focus on lesion areas compared to single-task networks. Chen et al. (17) developed a multi-task deep learning model for the differential diagnosis of non-small cell lung cancer using the TCIA database, achieving superior results (AUC: 0.843, 0.732) compared to both radiomics (AUC: 0.819) and single-task models (AUC: 0.788, 0.690). In another study, Gu et al. (23) demonstrated that a multi-task model effectively predicted the survival prognosis of locoregionally advanced nasopharyngeal carcinoma, with radiomic features from segmentation being independent prognostic factors. These studies align with our current research findings. Notably, in our study of 78 PET/CT cases (23 malignant), the prediction accuracy for malignant lesions was 65.22% (15/23). By comparing PET images with neural network heat maps, we found that the heat maps roughly encompassed areas of high uptake in about 11 cases. This insight could provide crucial guidance for biopsy or prostatectomy, although further research is needed.

In current clinical practice, MRI is the most common imaging modality for patients with suspected prostate cancer. However, it has limitations. A PI-RADS 3 score can make it challenging for clinicians to determine malignancy, and subtle cancerous lesions require interpretation by experienced clinicians, who are in short supply. Additionally, diagnostic variability among clinicians is a significant issue. The development of radiomics and artificial intelligence (AI) technologies is expected to address these challenges. Recently, Cai et al. (39) developed a multi-task deep learning model that effectively detects clinically significant prostate cancer using MRI. Trained on multi-center imaging data, it achieved excellent results in both internal (AUC = 0.89) and external validation sets (AUC = 0.86), demonstrating its potential to assist radiologists with high diagnostic sensitivity. Similarly, Debs et al. (40) used a 3D Unet model, showing that CNN-based detection models can outperform radiologists (TPRs = 0.93 vs 0.87). Meanwhile, research by Schrader et al. (41) confirmed that a modified Unet model could successfully segment and predict abnormal lesions on prostate MRI, significantly reducing unnecessary biopsies by approximately 49%. However, the multivariate logistic regression-based nomogram they used involved many variables, complicating its clinical implementation. Traditionally, CT has been excluded from essential examinations for predicting prostate cancer due to its low soft tissue resolution. However, recent studies have shown that radiomic features from CT can effectively predict bone metastasis in prostate cancer, achieving an accuracy of 0.9 in an independent test set. In our study, we used a multi-task deep learning model (MTDL) to predict prostate cancer based on non-contrast abdominal CT scans, achieving an AUC of 0.904 in the test set. This represents a new field compared to other AI studies using MRI, providing an important reference for early-stage prostate cancer diagnosis when patients often exhibit no clinical symptoms. CT is a relatively common and cost-effective imaging method that can be widely used for early detection. Additionally, the heatmaps generated by our model can indicate lesion locations. Although we also used radiomics models, their lower performance (AUC = 0.84) and limited interpretability have restricted their clinical application.

PSAD is recognized as a reliable predictor of clinically significant prostate cancer (4) (42), as demonstrated by Deniffel et al.’s study (3), which indicated that combining PSAD could significantly reduce unnecessary biopsies in patients with positive findings (PI-RADS>=3) on prostate MRI. By combining PSAD with PI-RADS scores, clinicians can better identify patients who require a biopsy. In our study, multivariate logistic regression across the training, test, and validation sets consistently identified PSAD as an independent predictive factor for clinically significant prostate cancer. Integrating PSAD with deep learning neural network recognition results could greatly enhance the detection rate of prostate cancer, reduce unnecessary biopsies, and importantly, both methods are cost-effective and easily accessible.

This study has several limitations. Firstly, the cases are from a single center, with a limited sample size and class imbalance, as positive samples account for only about one-third of the total. Future research should include data from multiple centers for a comprehensive comparative analysis to enhance the reliability of the results. Secondly, as this is a retrospective study, the data suffer from issues of completeness and homogeneity. Approximately 10% of patients lack data on C-reactive protein or alkaline phosphatase, which slightly affected the results. Lastly, this study focused solely on the application of CT in diagnosing prostate cancer and did not explore its role in clinical treatment and risk stratification. Clinical follow-up in future research is essential to provide clinicians with more valuable information.

## Conclusion

In summary, a multi-task deep learning neural network model can effectively predict clinically significant prostate cancer using abdominal CT. By incorporating PSAD and age into a nomogram, clinicians are provided with a non-invasive, cost-effective, and efficient method for prostate cancer detection.

## Data Availability

The raw data supporting the conclusions of this article will be made available by the authors, without undue reservation.
