# Supplementary material for: Significance of multi-task deep learning neural networks for diagnosing clinically significant prostate cancer in plain abdominal CT
Source: Front Oncol. 2025 May 2;15:1543230. doi: 10.3389/fonc.2025.1543230 (PMC12081240; doi:10.3389/fonc.2025.1543230)
Supplement: Supplementary file 1 [file DataSheet1.docx]

**Table S1** Detailed exclusion reasons and case distributions.

In this study, we included CT data from a total of 688 patients with suspected prostate cancer. However, only 539 cases were ultimately included in the final analysis, while 149 patients were excluded from the study for various reasons, They are:

| 1. The hip joint has a prosthesis, resulting in severe metal artifacts in the pelvis (N= 10) 2. Blurry CT images with low resolution (N=4) 3. Due to storage issues, some images were lost, and the obtained images do not show the complete prostate gland (N=17) 4. By reviewing the patient's medical history, it was found that the patient has a history of prostate surgery (N=14) 5. The patient is undergoing prostate endocrine therapy, castration therapy, or other medical treatments (N=30) 6. There are no biopsy results, or the clinical diagnosis is inconsistent with the biopsy results (N=14) 7. PSA values are missing, or the measurement of PSA levels was performed after the CT scan (N=38) 8. We did not obtain permission to access the patient's imaging data and related clinical information (N=22) |
| --- |

| Training group (N=276):  Non-csPCa (N=192):  BPH 54 cases,  Prostatitis 8 cases,  Prostatitis with BPH 128 cases,  Prostate cancer with ISUP < 2 2 cases;  csPCa (N=84):  Prostate cancer with Gleason score 7 20 cases,  Prostate cancer with Gleason score 8 29 cases,  Prostate cancer with Gleason score 9 20 cases,  Prostate cancer with Gleason score 10 15 cases; |
| --- |
| Test group (N=185):  Non-csPCa (N=111):  BPH 57 cases,  Prostatitis 7 cases,  Prostatitis with BPH 43 cases,  Prostate cancer with ISUP < 2 4 cases;  csPCa (N=74):  Prostate cancer with Gleason score 7 28 cases,  Prostate cancer with Gleason score 8 22 cases,  Prostate cancer with Gleason score 9 17 cases,  Prostate cancer with Gleason score 10 7 cases; |
| Validation group (N=78):  Non-csPCa (N=55):  BPH 30 cases,  Prostatitis 14 cases,  Prostatitis with BPH 11 cases,  csPCa (N=23):  Prostate cancer with Gleason score 7 3 cases,  Prostate cancer with Gleason score 8 6 cases,  Prostate cancer with Gleason score 9 11 cases,  Prostate cancer with Gleason score 10 3 cases; |

**Table S2** Details of the proposed work and previous works

In this supplementary table, we will list the details of our work (part A) and previous studies (part B).

**Part A**

| ***Step 1 : Literature review*** | After we clarified the research objectives, we began to review relevant literature, identify the main methods used, and collect clinical indicators mentioned in relevant literature. |
| --- | --- |
| ***Step 2 : Case Collection and Data Acquisition*** | We collected relevant medical images and clinical data based on the radiology and nuclear department's PACS system and also the hospital's medical record system. |
| ***Step 3 : Exclusion of Cases and Processing of Collected Data*** | After collecting relevant data, we will delete cases that do not meet the inclusion criteria. If the missing value of a clinical indicator reaches more than 10%, we will delete this indicator. If the missing value is less than 10%, we will use the average value to fill it. |
| ***Step 4 : Image Segmentation*** | Two clinicians from our department delineated the ROI from the CT using 3D-Slicer software. |
| ***Step 5 : Radiomics Feature Extraction*** | Based on the Pyradiomics package, we extracted 1155 radiomic features in each ROI for analysis. |
| ***Step 6 : Feature Selection and Modeling*** | We first compared the intraclass and interclass consistency coefficients (ICCs) of each radiomics features and deleted those features with ICCs less than 0.7. Then, we reduced the dimension of the features based on the Lasso algorithm and established a linear diagnostic model. |
| ***Step 7 : Code Implementation and Debugging*** | We developed the training process and architecture for single-task and multi-task neural network models using PyTorch. We ran the models and fixed any bugs we encountered. |
| ***Step 8 : Deep Learning Models Training*** | After debugging all the codes, we will train the model. We first train the single-task deep learning network model, followed by the multi-task deep learning network model. All training data comes from independent training sets. |
| ***Step 9 : Analysis of Clinical Indicators and Construction of Clinical Models*** | Univariate logistic regression was used to assess whether clinical indicators are independent predictors of clinically significant prostate cancer, and those indicators that were confirmed to be independent predictors in the training set were included in the establishment of the clinical model. |
| ***Step 10 : Comparison of Deep Learning Models*** | In this step, we compared the performance of the single-task deep learning network model and the multi-task deep learning network model on independent test sets and validation sets, and than selected the model with superior performance based on the model's diagnostic accuracy and Grad-cam heatmap analysis. |
| ***Step 11 : Development and Evaluation of Nomogram*** | In this stage, we combined the prediction results of the deep learning network model with superior performance and the clinical indicators selected in step 9 to develop a Nomogram model. |
| ***Step 12 : Comparison and Statistical Test of Different Models*** | We comprehensively compared and analyzed the prediction results of the radiomics model, clinical model, single-task deep learning network model, multi-task deep learning network model, and Nomogram model, and Delong test was used to compare the differences in the AUC values amomg the models. |
| ***Step 13 : Draw Conclusions and Create Tables*** | Finally, based on the statistical results of the previous step, we summarized the results in the form of a table and discussed the final conclusions within the research group. |
| ***Step 14 : Discussion of Results and Manuscript Writing*** | After discussion, we came to the final research results and wrote the experimental process and all the results into this paper. |

**Part B** In this section, we mainly summarize and compare 4 previous papers that are similar to our research

| **Aspect** | **Propoesd Work** | **Previous Work 1[1]** | **Previous Work 2[2]** | **Previous Work 3[3]** | **Previous Work 4[4]** |
| --- | --- | --- | --- | --- | --- |
| **Objective** | Prostate Cancer Diagnosing in abdominal CT | Bladder cancer muscle invasion prediction in MRI | Prognostic  prediction in nasopharyngeal carcinoma on PET/CT | Prostate cancer detection on MRI | Prostate cancer risk assessment on MRI |
| **Methodology** | 3DUnet modified multi-task deep learning network | ResNet50 modified multi-task deep learning model | Unet-based segmentation backbone and DenseNet-based survival network | two-stage cascaded Unet model (Segmentation and Detection) | Modified nnUNet models |
| **Data used** | 539 | 121 | 886 | 2734 | 1627 |
| **Performance (AUC)** | 0.904, 0.864 | 0.932 | 0.750, 0.702 | 0.849 (Patient level) | 0.889 |
| **Key finding** | Abdominal non-contrast CT can effectively detect prostate cancer based on multi-task deep learning model. | MRI images based on T2WI can predict muscle invasion in bladder cancer. | Nomogram model based on multi-task deep learning can effectively predict the prognostic survival of nasopharyngeal carcinoma | Incorporating regional segmentation can more effectively increase the detection rate of prostate cancer by DL models. | The nnUNet neural network model can make the prediction of Risk calculators more stable, avoiding approximately 49% of unnecessary biopsies. |
| 1. Li, J., et al., *Predicting muscle invasion in bladder cancer based on MRI: A comparison of radiomics, and single-task and multi-task deep learning.* Comput Methods Programs Biomed, 2023. **233**: p. 107466.  2. Gu, B., et al., *Multi-task deep learning-based radiomic nomogram for prognostic prediction in locoregionally advanced nasopharyngeal carcinoma.* Eur J Nucl Med Mol Imaging, 2023. **50**(13): p. 3996-4009.  3. Hosseinzadeh, M., et al., *Deep learning-assisted prostate cancer detection on bi-parametric MRI: minimum training data size requirements and effect of prior knowledge.* Eur Radiol, 2022. **32**(4): p. 2224-2234.  4. Schrader, A., et al., *Prostate cancer risk assessment and avoidance of prostate biopsies using fully automatic deep learning in prostate MRI: comparison to PI-RADS and integration with clinical data in nomograms.* European Radiology, 2024. **34**(12): p. 7909-7920. | | | | | |

Fig. S1 training-validation curves


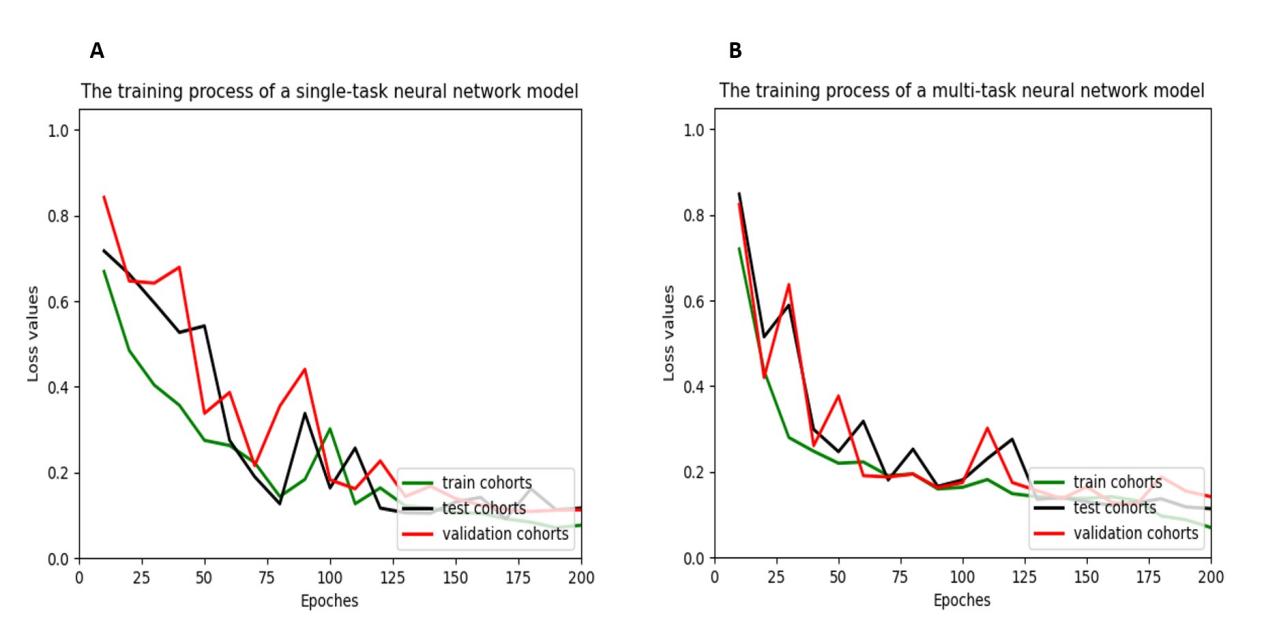


Fig.S1 Training-validation curves for the single-task and multi-task deep learning models are presented, with the X-axis representing the number of iterations and the Y-axis depicting the loss function.

Fig.S2 heatmap of single-task and multi-task deep learning


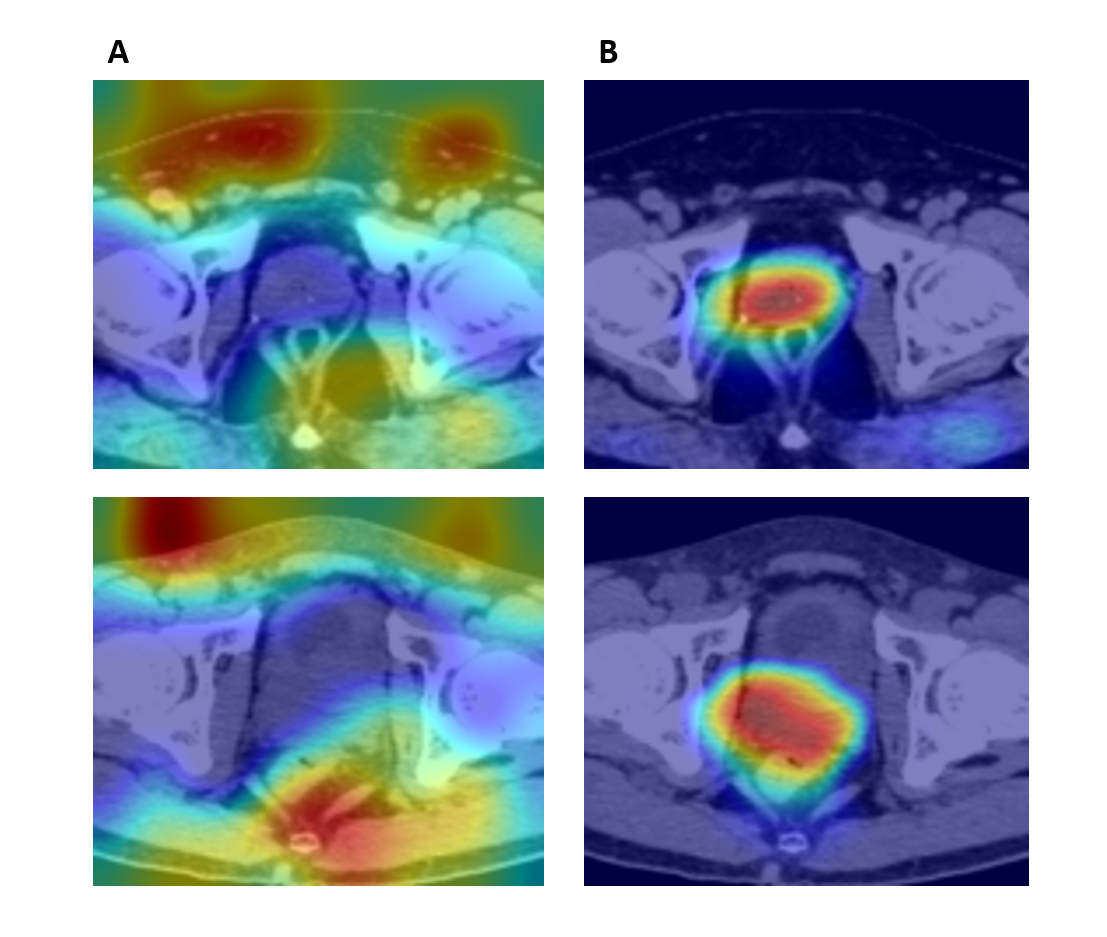


Fig.S2 Comparison of Grad-CAM Heatmaps. Group A displays the output from the final convolutional layer of a single-task neural network, while Group B shows the output from a multi-task neural network. In the images, it is clear that the single-task model focuses on areas distant from the prostate tissue, making its inference less reliable for clinicians. In contrast, guided by the segmentation task, the multi-task model successfully concentrates on the prostate, aiding in the analysis of pathological changes and providing essential reference points for diagnosing clinically significant prostate cancer.


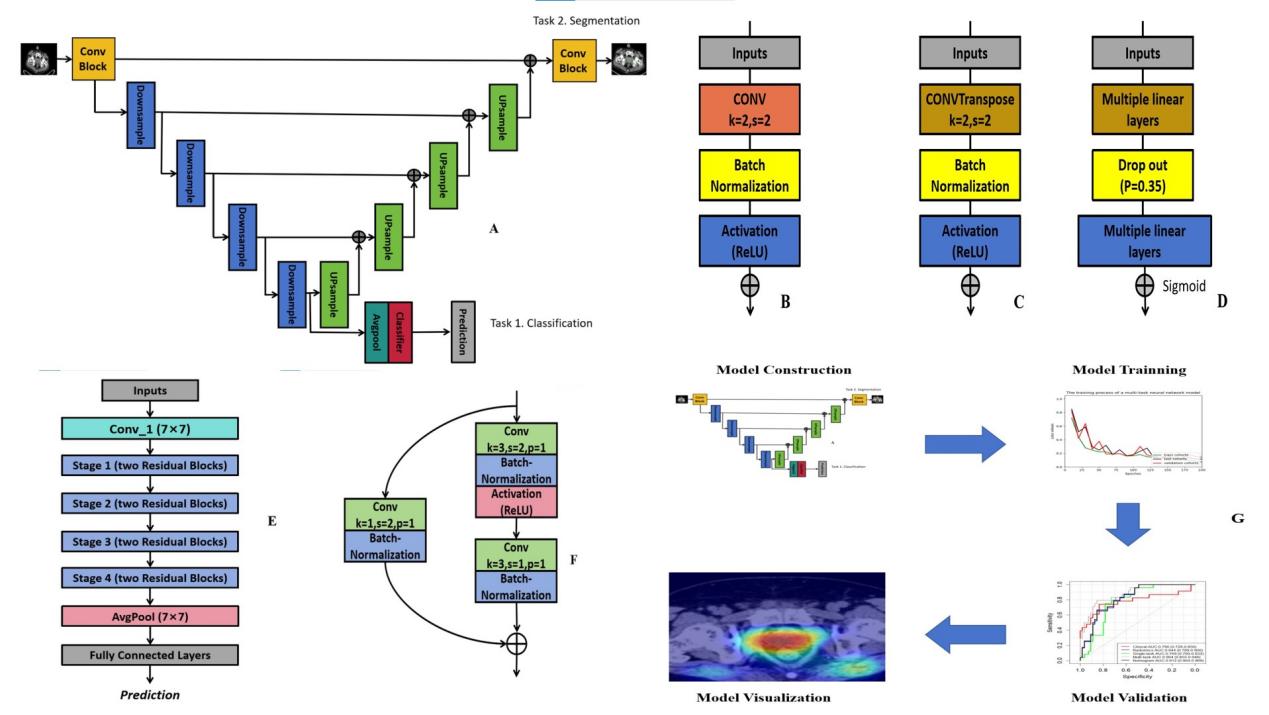


Fig.S3 Network Architecture of Deep Learning Models. Figure A illustrates the multi-task deep learning network model we studied. Below it, Figures B, C, and D represent the downsampling module, upsampling module, and classification module within Figure A, respectively. Figures E and F in the last row depict the single-task deep learning network model (ResNet18), with Figure E showing the overall structure and Figure F detailing the residual block. Figure G represents the general training process of the neural network model.
